# *Eucommia ulmoides* Oliver repairs the disorder of intestinal microflora caused by high starch in *Micropterus salmoides* and improves resistance to pathogens

**DOI:** 10.3389/fmicb.2023.1223723

**Published:** 2023-09-21

**Authors:** Hongli Liu, Fulong Li, Hong Tang, Baipeng Chen, Yi Geng, Defang Chen, Ping Ouyang, Liangyu Li, Xiaoli Huang

**Affiliations:** ^1^Department of Aquaculture, College of Animal Science & Technology, Sichuan Agricultural University, Chengdu, Sichuan, China; ^2^Fisheries Research Institute, Chengdu Academy of Agriculture and Forestry Sciences, Chengdu, Sichuan, China; ^3^Department of Basic Veterinary, College of Veterinary Medicine, Sichuan Agricultural University, Chengdu, Sichuan, China

**Keywords:** *Eucommia ulmoides* Oliver, *Micropterus salmoides*, intestinal microflora, starch, immune

## Abstract

*Eucommia ulmoides* Oliver (EuO) is a natural medicine that can improve the composition of intestinal flora in fish, but more experiments and data are needed to support whether it can effectively improve the changes of intestinal flora and intestinal damage caused by high starch. This study examined the changes in intestinal structure as well as intestinal flora before and after the addition of EuO to high-starch diets and analyzed the effects of such changes on immune and digestive functions. The results showed that EuO reduces mortality during *Nocardia seriolae* attack and can reduce starch-induced intestinal inflammation. *Eucommia ulmoides* Oliver supplementation was able to alter the changes of intestinal flora in fatty acid degradation, bacterial chemotaxis, porphyrin metabolism and flagella assembly caused by high starch. By analyzing the abundance and correlation of bacterial communities, three bacterial communities that were significantly related to the intervention effect of EuO were screened. Further analysis revealed that EuO supplementation reduced the increase in abundance of *Limnochordaceae*, *Nitrolancea*, *Lysinibacillus*, and *Hydrogenispora* induced by high starch, which were negatively correlated with levels of the immunoreactive substance LZM in fish. This study reveals the regulatory effects of EuO on the intestinal flora of *Micropterus salmoides* fed on high starch diets, and provides a theoretical basis for reducing starch damage to fish in production.

## 1. Introduction

The intestine is one of the most important internal organs of aquatic vertebrates, which not only has the functions of digestion and absorption, but also involves regulating immunity and balancing metabolism (Rombout et al., 2011). The intestine hosts a variety of microorganisms, forming a complex and diverse intestinal microflora. There is a close interaction between the intestinal flora and aquatic vertebrates, which not only help the host to resist the adverse external factors, but also regulates the immune processes of the host (Zhou et al., 2020). Intestinal flora and its products influence the immune function of aquatic vertebrates in several ways. On the one hand, specific probiotics promote the development and maturation of intestinal mucosa-associated lymphoid tissue, thereby enhancing mucosal barrier function and local immune response (Maynard et al., 2012). On the other hand, metabolites produced by intestinal flora are able to bind to pattern recognition receptors (e.g., TLR and NLR), thereby activating signaling pathways and transcription factors (e.g., NF-κB and AKT2), regulating the expression of cytokines and antimicrobial peptides, and enhancing resistance to pathogen invasion (Ni et al., 2017). In addition, there is also competitive exclusion between intestinal flora that facilitates the inhibition of pathogenic bacterial growth. For example, anaerobic bacteria inhibit the colonization of conditionally pathogenic bacteria by producing acidic metabolites that create a low pH microenvironment in the intestine (Goldstein et al., 2015). However, the intestinal microflora is vulnerable to external disturbances, such as invasion of pathogens, use of antibiotics, and changes in feed composition, resulting in an imbalance of intestinal flora, immune dysfunction, and ultimately endangering the health of aquatic vertebrates (Rombout et al., 2011).

Starch is the main source of carbohydrates in animal feeds, providing energy for animal growth, and it can be used as an inexpensive ingredient in feeds, effectively reducing feed costs (Li et al., 2020). However, carnivorous fish (e.g., *Micropterus salmoides*) use starch less efficiently, and excessive intake of dietary starch leads to slow growth, reduced immunity and increased mortality, so the starch content in carnivorous fish feed needs to be moderately controlled (Lin et al., 2018). Moreover, high starch intake can also disrupt the balance of fish gut microorganisms, which in turn affects the health of fish. Studies have shown that excessive starch intake can cause a decrease in the number of beneficial bacteria in the gut of largemouth bass and increase the risk of inflammation in the intestine (Huang X. et al., 2021). Despite these hazards of starch, its use cannot be avoided in order to balance feed costs and profits. Therefore, there is a need to find an effective medicine to reduce the health risks associated with high starch feeds.

*Eucommia ulmoides* Oliver (EuO) belongs to the *Eucommia* Oliv in the Eucommiaceae (Huang L. et al., 2021). As an herbal feed additive, it has the effect of enhancing the immunity of animals and is widely used in the feed industry. With the discovery of pharmacological components of EuO, more and more people pay attention to it (Huang L. et al., 2021). Studies have shown that flavonoids, chlorogenic acid and other components in EuO have significant effects on antioxidant damage, treatment of hyperlipidemia and hyperglycemia (He et al., 2014). In addition, EuO has a regulatory effect on intestinal microecology. The latest research have found that *Eucommia ulmoides* leaf extract could alleviate the intestinal damage caused by high-fat diet and improve the intestinal flora structure of *Ictalurus punctatus* (Zhang et al., 2022). Moreover, it was also found in a study on *Carassius auratus* that *EuO* extract could improve the intestinal flora structure and increase the activity of non-specific immune enzymes in the intestinal and liver tissues (Jin et al., 2022). However, there is a lack of studies on whether EuO can alleviate the effects of high-starch diets on the intestinal flora of *M. salmoides*. Therefore, in this study, we investigated whether EuO has the function of alleviating intestinal tissue damage caused by high starch levels, and revealed the microbial mechanism of EuO protection in the intestine.

## 2. Materials and methods

### 2.1. Diet formulation

The experimental diets, namely the control diet containing 13 g/kg α-cassava starch and the starch diet containing 224 g/kg α-cassava starch, were prepared and stored in the same way as in our previous study (Huang X, et al., 2021), and the diet formula and proximate composition analyses as shown in Supplementary Table S1. Dietary protein sources included fish meal, casein, and soy protein concentrate, while dietary lipid sources included soybean oil and soy lecithin. During the production process, all feed ingredients were accurately weighed, mixed proportionally, pelleted into 2.0 mm pellets using a pelleting machine, and stored in sealed plastic bags at −20°C. The determination of feed ingredients was consistent with the previous study (Huang X, et al., 2021).

### 2.2. Extract preparation

The method of EuO extraction was consistent with our previous study (Liu et al., 2023). Briefly, EuO was homogenized into powder and after three extractions with ethanol, the extracts were evaporated and concentrated using a rotary evaporator (IKA RV10, Germany) and circulating water pump (YUHUA, China), and then the concentrates were dried and assayed for flavonoid content by a flavonoid assay kit (Nanjing Jiancheng Bioengineering Institute, Nanjing, China), which measured 6.45 mg g^−1^. 30 mg kg^−1^ EuO extract was added to the starchy diet as EuO diet.

### 2.3. Fish and experimental treatment

*Micropterus salmoides* were obtained from local fisheries (Sichuan China). Before starting the experiment, fish were reared in the experimental environment and fed with the control diet for 1 weeks. During this period, we refreshed a third of the rearing water every 3 days and determined the water quality parameters before and after refreshing the water. The water temperature was 25 ± 2°C, pH was 7–8, photoperiod was 12 h light: 12 h dark, and the control feed was fed at 3% of fish body weight during the period, with feeding times of 9:00 and 18:00 every day. The *M. salmoides* were not fed 1 d before the start of the experiment, and healthy, uninjured and robust *M. salmoides* (weight: 12.5 ± 0.7 g) were selected for this experiment.

After the temporary feeding, the 360 *M. salmoides* were randomly and equally divided into 12 glass tanks (*n* = 30), and divided into two groups (Z group with three tanks and GT group with nine tanks). The Z group was fed the control diet and the GT group was fed the starch diet (Supplementary Table S1). Nine samples were taken from each group after 30 d of continuous feeding to determine the corresponding indices.

After the above 30 d breeding experiment, *M. salmoides* in the GT group were further divided equally into the G1, G2, and GT group (*n* = 81). The G1 group was fed the EuO diet on a cyclic basis (7 days feeding and 7 days stopping with two consecutive cycles), while the G2 group was continuously fed the EuO diet. Meanwhile, group Z, which was fed the control diet, was used as the control.

### 2.4. Challenge test

After the feeding experiment, 12 *M. salmoides* in each group were selected and injected intraperitoneally with 0.2 mL of 1 × 10^7^ CFU/mL *Nocardia seriolae*. The experimental conditions were the same as those during the feeding experiment, and were observed for 18 days. During the experiment, the death of fish in each group were recorded, and the survival rate of each group were calculated. In addition, anatomical observations of dying fish were carried out.

### 2.5. Isolation and identification of strains

The dying *M. salmoides* were selected after the challenge, disinfected with 75% ethanol on its body surface, and dissected aseptically. Under aseptic conditions, spleen, liver, and kidney tissues were inoculated on BHI using the plate streaking method and incubated at 28°C for 7d. And then, single colonies were picked and placed in BHI liquid medium for amplification. The bacterial genome was extracted using the Bacterial Genomic DNA Extraction Kit (Foregene, Shanghai, China), and used as a template for PCR amplification with primer 27\u00B0F/1492 R. Sequences were compared with NCBI database sequences, and the most similar bacterial sequences were selected. MEGA11 was used for sequence alignment, and the Neighbor-Join Method was used to construct a phylogenetic tree with a bootstrap test of 1,000 repetitions.

### 2.6. Sample collection

The fish were sampled after 30 d and 60 d of the feeding trial. All fish were anesthetized with MS222 before sampling. At 30 and 60 days, 9 *M. salmoides* were randomly selected from each group and their intestines were collected for histological observation. In addition, another nine fish in each group were selected at 60 days to collect whole blood and intestine. Whole blood were collected using a 1 mL syringe containing sodium heparin. The whole blood was centrifuged at 4°C for 10 min at 3000 rpm to collect plasma. The plasma of three fish is pooled together. Intestinal samples were placed in sterile petri dishes, and about 2 cm of the midgutsegment was cut with sterile dissecting scissors, then the intestinal walls were dissected longitudinally and the intestinal contents were scraped and placed in 2.0 mL centrifuge tubes. To reduce inter-individual variation, the intestinal contents and intestines of three fish from each tank were pooled for microbiota analysis and enzyme activity and relative mRNA expression measurements, respectively.

### 2.7. H&E staining

The collected intestinal tissues were fixed in 10% neutral formalin, and the fixed tissues were subsequently trimmed into blocks and dehydrated with graded ethanol, transparent in xylene, embedded in paraffin, sectioned at 4 μm, stained with H&E, and finally observed under light microscopy (Nikon Eclipse 50i, Nikon, Tokyo, Japan).

### 2.8. Enzyme activity assay

Plasma levels of lysozyme (LZM) were measured using kits (Nanjing Jiancheng Bioengineering Institute, Nanjing, China) following the manufacturer’s instructions. LZM were measured by the colorimetric method. The activity of three enzymes in intestinal tissue was measured according to the instructions provided in the α amylase, lipase, and trypsin kits (Nanjing Jiancheng Bioengineering Institute, Nanjing, China). α amylase was measured by starch-iodine colorimetry, lipase by colorimetry, and trypsin by ULTRAVIOLET colorimetry.

### 2.9. Extraction of bacterial genome and high throughput sequencing of microbial 16S rRNA

Samples of intestinal contents were collected using a bacterial DNA isolation kit (Foregene, Chengdu, China), and each DNA sample was tested for quality and integrity using a 1% agarose gel. PCR amplification of the variable regions V3 to V4 was performed using primers 338-F (5′ ACTCCTACGGGAGGCAGCAG 3′) and 806-R (5′ GGACTACHVGGGTWTCTAAT 3′). The PCR products were mixed in equal amounts according to the PCR product concentrations, and after sufficient mixing, the PCR products were detected by agarose gel electrophoresis with a mass fraction of 2%, and the target bands were subjected to gel recovery using the QIAquick Gel Extraction Kit (Qiagen, Germany). Quantification was performed using QuantiFluorTM-ST Blue Fluorescence System (Promega, Beijing, China). Sequencing libraries were constructed and high-throughput sequencing was performed on the purified samples. The raw data were spliced using FLASH software (version 1.2.11), the spliced sequences were quality filtered by Trimmomatic software (version 0.33), UCHIME software (version 8.1) identifies and removes chimeras. Clustering was performed using USEARCH (version 10.0) at a 97% similarity level to obtain operational taxonomic units (OTUs). Microbial diversity analysis was performed based on the MegiCloud platform to obtain α-diversity index, β-diversity, species annotation and taxonomic analysis.

### 2.10. Co-occurrence networks

Based on Klaus Schlaeppi’s method, we conducted network analysis on the data (Hartman et al., 2018). Briefly, two-by-two Spearman correlation calculations of OTUs were performed based on the TMM standardized OTU table. Paired OTUs with Spearman’s *r* > 0.6, *p* < 0.001 were selected for correlation network analysis and visualized using the Fruchterman-Reingold layout. At the same time, in order to determine the network module, the greedy optimization modular algorithm is used for clustering.

### 2.11. Quantitative real-time PCR (qRT-PCR) analysis

Measurements of relative mRNA expression included total RNA extraction from intestinal tissues, reverse transcription to cDNA, and qRT-PCR analysis. Our previous study (Liu et al., 2023) provides more detailed information. The specific primer sequences used in this study were designed with reference to previous studies (Gu et al., 2022; Yu et al., 2022; Liu et al., 2023) and are shown in Table 1. *β-actin* was selected as the reference gene, and the relative expression was calculated using the 2^-∆∆ct^ method.

**Table 1:** Primer sequences used for real-time PCR analysis.

Gene	Reference	Primers
*TNF-α*	XM_038710731.1 (Gu et al., 2022)	F_ CTTCGTCTACAGCCAGGCATCG R_ TTTGGCACACCGACCTCACC
*IL-10*	XM_038696252.1	F_ CGATTCTGCCAACAGCCTTG R_ GCTCGTCGAAGATCTGCTGT
*NF-κB*	XP_027136364.1 (Yu et al., 2022)	F_CCACTCAGGTGTTGGAGCTT R_TCCAGAGCACGACACACTTC
*β-actin*	XM020651307.1 (Liu et al., 2023)	F_ AAAGGGAAATCGTGCGTGAC R_ AAGGAAGGCTGGAAGAGGG

### 2.12. Statistical analysis

Data were analyzed using one-way analysis of variance (ANOVA), Duncan method was used for multiple comparison. Before applying one-way ANOVA, all data were systematically tested for normality and homogeneity. All data are expressed as mean ± standard deviation. A value of *p* < 0.05 was considered significant. Detailed information is shown in the figure legend.

## 3. Results

### 3.1. Therapeutic effect of *Eucommia ulmoides* Oliver on intestinal injury induced by high starch

**Figure 1:**
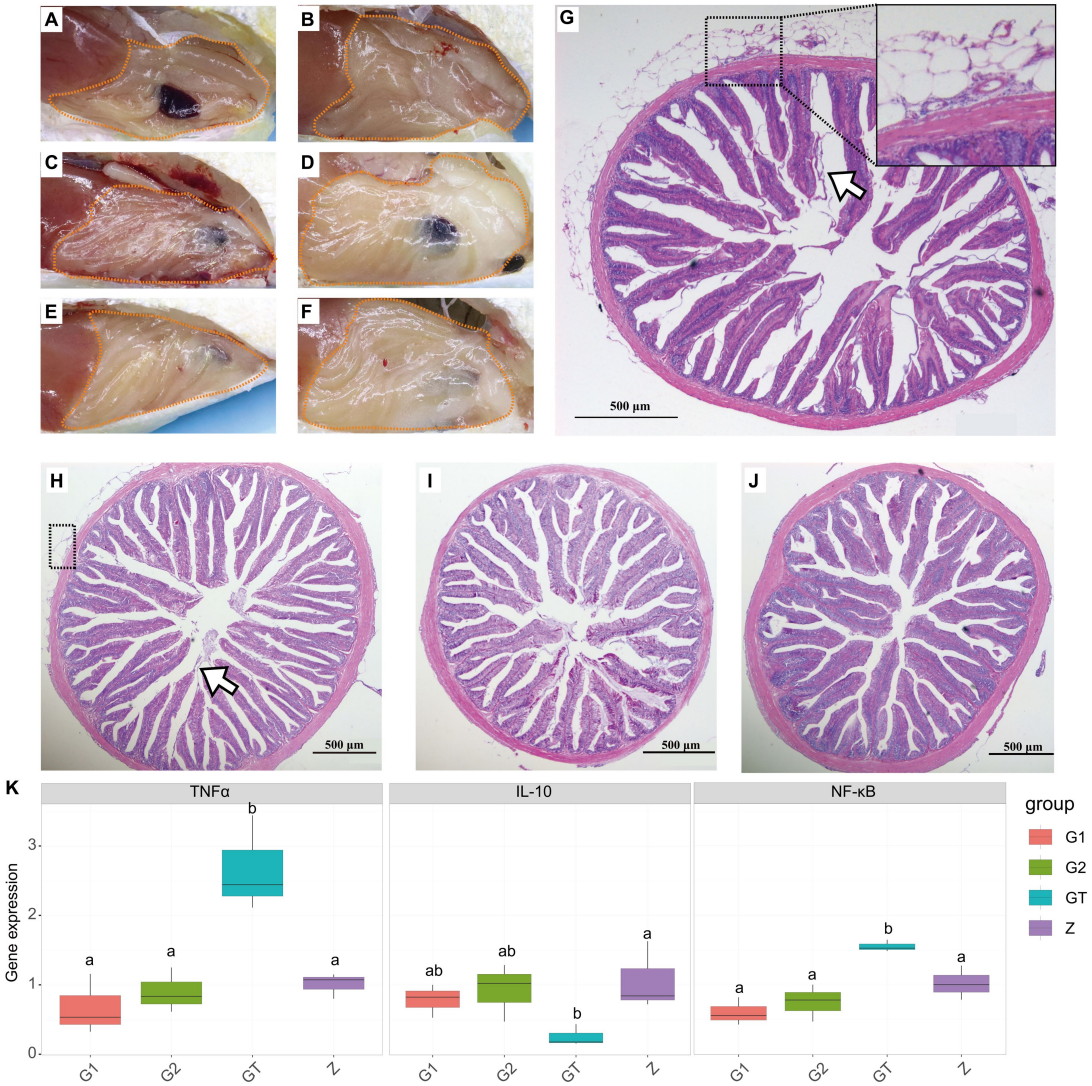
Peripheral fat accumulation in the intestine and histological changes of *Micropterus salmoides* intestine. **(A–F)** Gross intestinal pathology in different groups of *M. salmoides*, fat accumulation (dotted). **(A,B)** Z group and GT group were fed high starch diet for 30 days, respectively. **(C–F)** Group Z, GT, G1 and G2 were fed with high starch diet for 60 days, respectively. **(G–J)**: HE staining of intestinal tissues of different groups of *M. salmoides*. **(G)** GT group fed high starch for 60 days, serosal layer vacuolation and inflammatory cell infiltration (dotted), striate border detachment (arrow). **(H)** GT group fed high starch for 30 days, serosal layer vacuolation and inflammatory cell infiltration (dotted), striate border detachment (arrow). **(I,J)** The G1 and G2 groups were fed *Eucommia ulmoides* Oliver (EuO) at intervals and continuously, respectively. **(K)** mRNA expression of *TNFα, IL-10 and NF-κB* in each group. *p* < 0.05 with differently labeled letters.

To characterize the efficacy of EuO on intestinal damage caused by high starch, fish intestines were observed and stained with H&E and gene expression detection. After feeding the high starch diet for 30 days, the amount of fat deposition around the intestine in the GT group of *M. salmoides* was higher than that in the Z group (Figures 1A,B). In addition, HE staining showed that the serosal layer of the intestine was detached and necrotic, and the structure of the intestinal epithelium was disordered as well as the striated border was detached (Figure 1H). When the high starch diet was fed for 60 days, the fat accumulation in the peripheral intestine of the GT group increased and the serosal layer of the intestine became severely vacuolated, and inflammatory cell infiltration was found in the serosal and muscle layers (Figures 1C,D,G). In addition, the intestinal epithelial cell structure was more disturbed and a large number of striate borders were detached compared to the 30-day GT group (Figures 1G,H). However, when EuO was added to the high-starch diet, the fat accumulation around the intestine of the *M. salmoides* was reduced (Figures 1E,F) and the symptoms of intestinal serosal layer and striate borders detachment were improved (Figures 1I,J). In the gene expression assay, it was found that the expression of *NF-κB* and *TNFα* in the starch group was significantly higher than that in the control group, while the expression of *IL-10* was decreased. With the addition of EuO, the expression of *NF-κB* and *TNFα* genes decreases significantly and tends to be similar to that of the control group, whereas the expression of *IL-10* is increased compared to that of the starch group (Figure 1K).

### 3.2. *Eucommia ulmoides* Oliver increased resistance of *Micropterus salmoides* to pathogens

**Figure 2:**
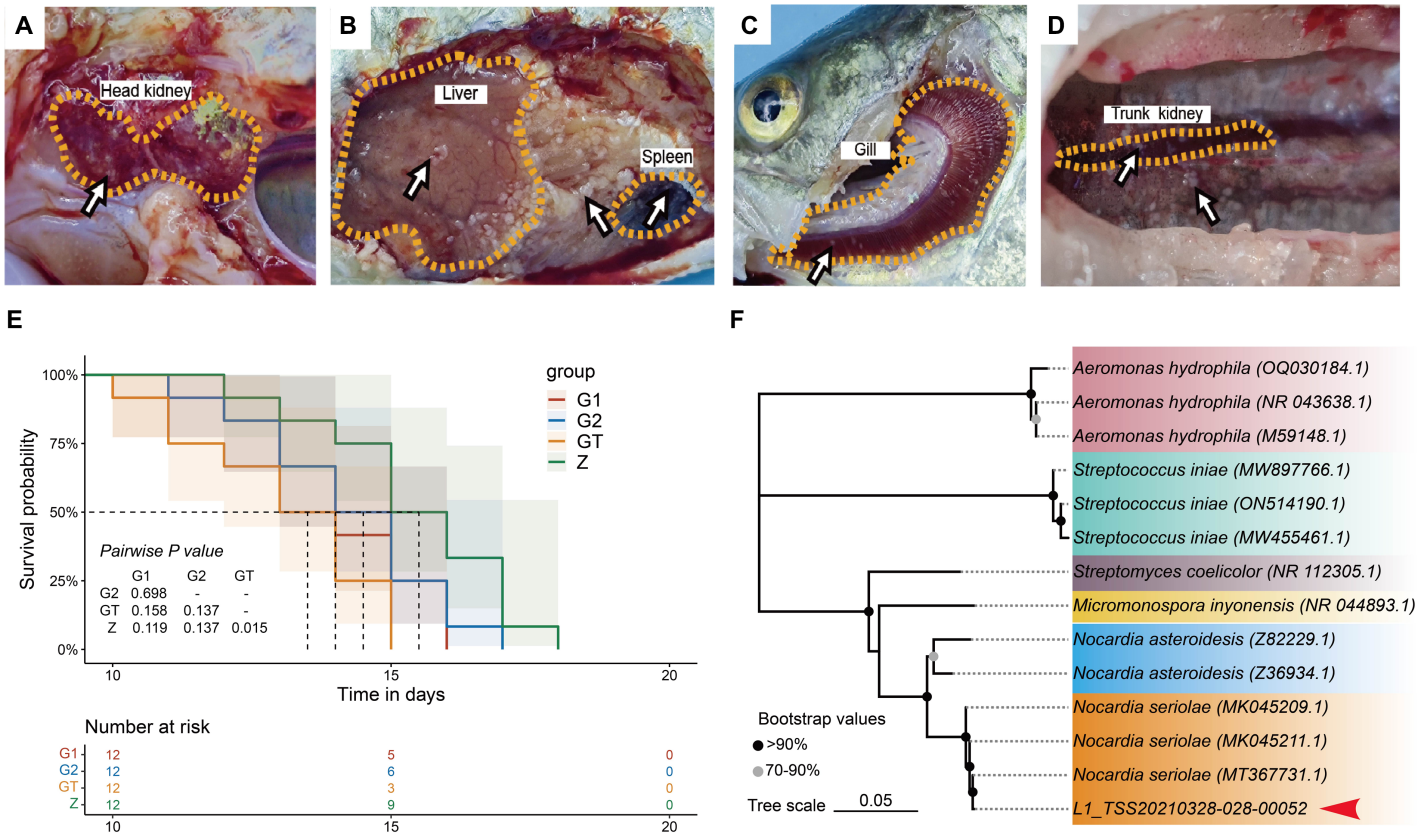
Lesions in various groups of *M. salmoides* after *Nocardia seriolae* attack. Nodules (arrows). **(A)** Z group. **(B)** GT group. **(C)** G1 group. **(D)** G2 group. **(E)**: Survival curve showed the survival rate of each group of *M. salmoides* after *N. seriolae* challenge. **(F)** Construction of a phylogenetic tree of liver isolates using the neighbor-joining method. The accession number of the selected strains in NCBI is indicated in parentheses. The sequences to be determined in this study are marked with red arrowhead.

High starch can damage intestinal barrier function and enhance pathogen susceptibility. In order to verify whether EUO enhances resistance to pathogens, we conducted a challenge experiment on *M. salmoides*. The results showed that obvious nodules appeared in liver, spleen, kidney, gills and head kidney of dying fish in each group (Figures 2A–D). And the survival rate of the GT group was significantly lower than that of the Z group. However, the survival rate of the G1 and G2 groups with the addition of EuO was improved and approached that of the Z group (Figure 2E). In addition, *N. seriolae* was also isolated from the liver of the dying fish (Figure 2F).

### 3.3. *Eucommia ulmoides* Oliver changes intestinal microbial diversity of *Micropterus salmoides*

A total of 581,007 valid sequences, with an average length of 424 bp, were obtained from the four groups of samples by sequencing the 16S rRNA of the intestinal microorganisms of *M. salmoides*. As shown by the dilution curve in Figure 1A, the number of extracted sequences reached more than 40,000, and the curve tended to be flat, indicating that the data sequencing of each group was reasonable, and that the current sequencing depth of each group was sufficient to reflect the microbial diversity contained in the community sample (Supplementary Figure S1A). The Rank-abundance curve in Figure 1B shows that the trend of decreasing abundance tends to be flat as the OTU Level Rank increases, indicating that the uniformity and abundance of species in the samples are good and can be used for subsequent analysis (Supplementary Figure S1B).

**Figure 3:**
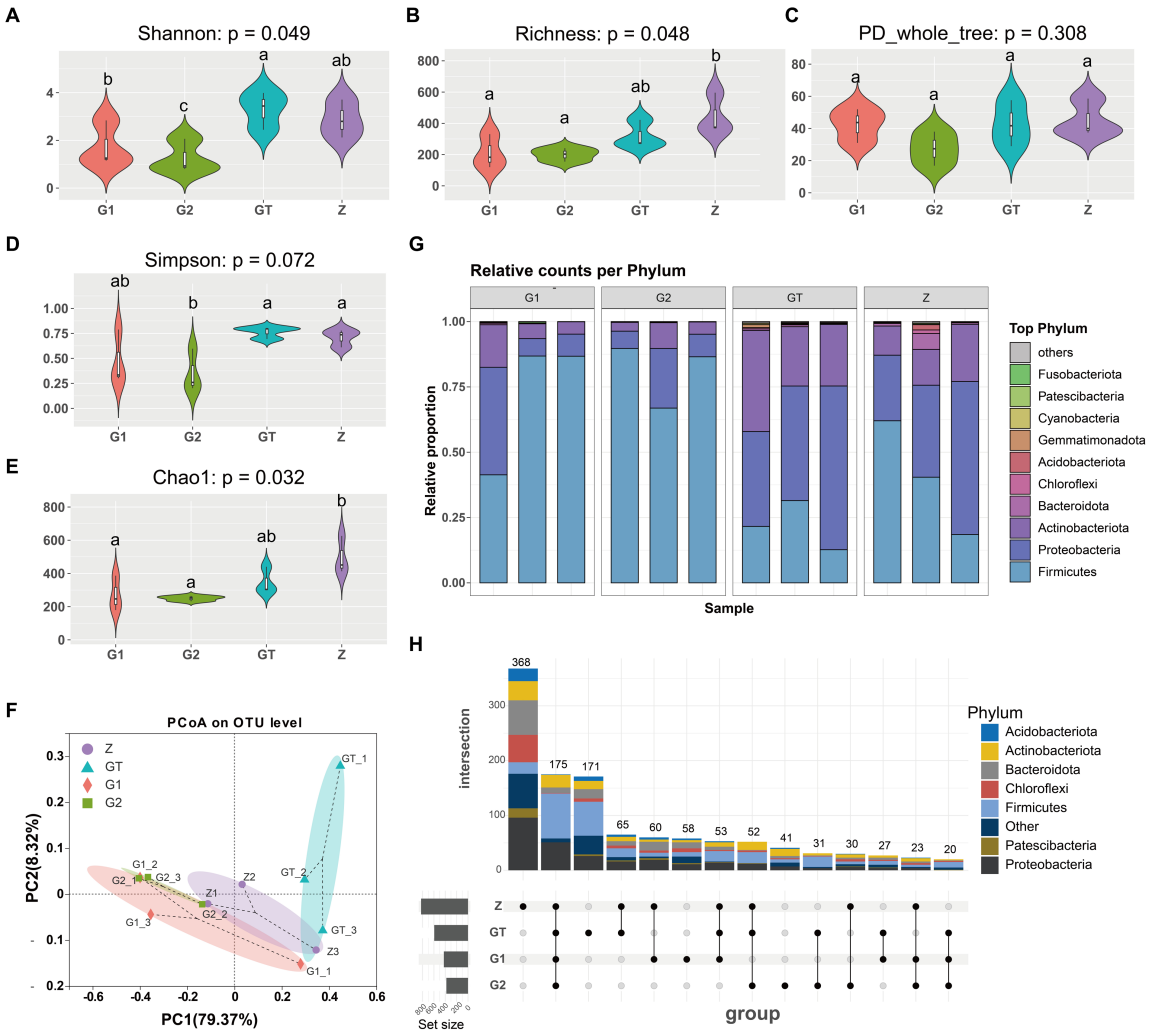
Intestinal microbial diversity analysis. **(A–E)** Effect of different treatments on Shannon, Richness, PD_whole_tree, Simpson and Chao1 of intestinal microorganisms. **(F)** Principal coordinates analysis (PCoA) of samples. **(G)** Species analysis of each sample at phylum taxonomic level. **(H)** Analysis of OTUs specific to each group of intestinal microorganisms. The analysis of OTUs specific to each group of intestinal microorganisms, the horizontal bar (left part) represents the number of OTUs contained in each group without duplication, the vertical bar (upper right part) is the number of OTUs unique and common to each group, and below the vertical bar is the classification of OTUs unique and common to each group, corresponding to the bars in the upper part, and the connecting line represents common. *p* < 0.05 with differently labeled letters.

Microbial community Alpha diversity used to analyze the effect of EuO on the intestinal microbial diversity of *M. salmoides*. Calculation of colony diversity index (Shannon, Simpson, Richness, PD_whole_tree, Chao1) at 97% sequence similarity level. After 60 days of breeding experiments, it was found that there was no significant difference in the α-diversity of the intestinal flora between the high starch group and the control group, but the addition of EuO inhibited the growth of certain bacteria, leading to a decrease in the α-diversity of the bacterial flora (Figures 3A–E). Based on the phylum taxonomic level, the Actinobacteriota, Proteobacteria and Firmicutes were found to be the main constituent phylum of intestinal microorganisms in each group of *M. salmoides*. Compared with the Z group, the GT group showed a decrease in the Firmicutes and an increase in the Proteobacteria and Actinobacteriota. However, after feeding EuO, the Firmicutes increased and the Proteobacteria and Actinobacteriota decreased in the G1 and G2 groups compared to the GT group (Figure 3G). Beta diversity of microorganisms was then analyzed by PCoA, and it was found that at the OTU level, principal component 1 (PcoA1) and principal component 2 (PcoA2) explained 79.37 and 8.32% of the variance, respectively, with a cumulative explanatory power of 87.69% (Figure 3F). Differentiation was observed among different sample groups, with significant differentiation in the G2 and GT groups (Figure 3F). Further analysis of the specificity of intestinal microbe OTU in each group showed that there were 368 OTU unique to group Z, 171 OTU unique to group GT, 58 OTU unique to group G1, 41 OTU unique to group G2, and 175 OTU common to the four groups. And the OTU of these four groups are mainly concentrated in Firmicutes, Proteobacteria and Actinobacteriota. In addition, the number of OTUs shared by Z and GT was 65, the number of OTUs shared by G1 and GT was 27, the number of OTUs shared by G2 and GT was 31, and the OTUs shared by G2 and GT were mainly concentrated in Firmicutes (Figure 3H).

### 3.4. Differences in composition and function of intestinal microbiota

**Figure 4:**
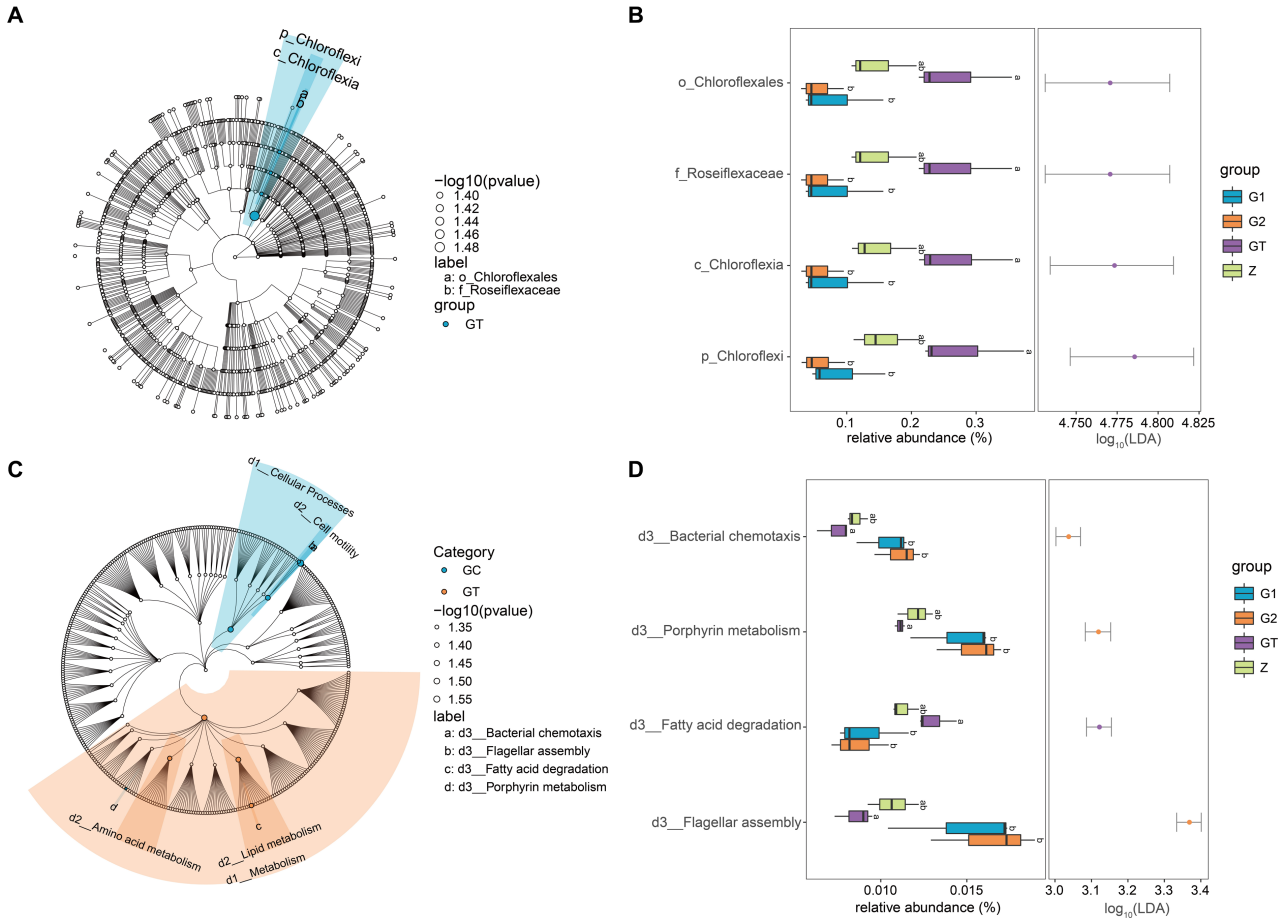
Differential analysis of intestinal microorganisms and kegg pathway among groups. **(A)** LEfSe analysis of microorganisms. Small circles: The circles radiating from the inside out represent the classification level from phylum to genus. The diameter of the small circles represents the relative abundance. Color: the species with no significant differences were uniformly colored white, and the species with significant differences were colored with the group. The species name of the Biomarker that is not be shown is displayed on the right, and the letter number is the same as that in the figure. **(B)** Difference analysis of LEfSe screened microorganisms among groups. **(C)** LEfSe analysis of kegg pathway. **(D)** Difference analysis of LEfSe screened keg pathway among groups. *p* < 0.05 with differently labeled letters.

In order to further study the effect of EuO on microbial and kegg pathway, LEfSe analysis was used. The LEfSe analysis (LDA threshold of 4) revealed that Roseiflexaceae, Chloroflexales, Chloroflexia, and Chloroflexi would be slightly elevated in the GT group compared to the control group, but the addition of EuO would significantly reduce their abundance (Figures 4A,B). Subsequently, the prediction of gene function (LDA threshold of three) showed that the Fatty acid degradation in GT group was slightly higher than in the control group. The Bacterial chemotaxis, Porphyrin metabolism and Flagellar assembly were slightly lower than in the control group, while the G1 and G2 groups showed the opposite trend to the GT group (Figures 4C,D).

### 3.5. *Eucommia ulmoides* Oliver regulates the abundance of immune-related bacteria

**Figure 5:**
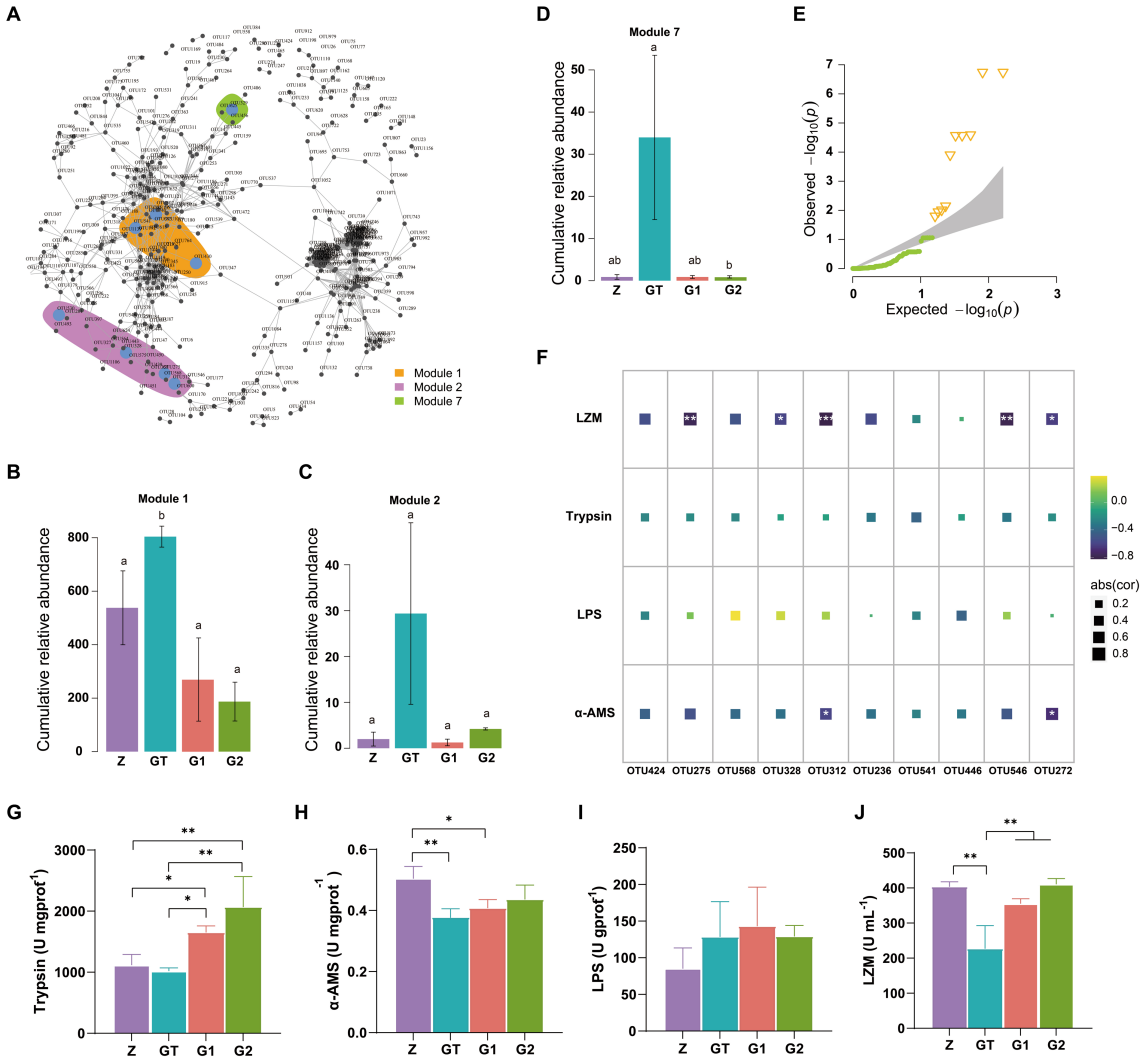
OTUs co-occurrence network. **(A)** Cooccurrence network of significant correlation among OTUs in each group (Modularization in igraph using fast greedy algorithm, TMM normalized CPM counts, Spearman’s *r* > 0.6, *p* < 0.001). **(B–D)** The relative abundance of major modules (Module1, Module2, and Module7) in the microbial network containing the OTU of intergroup differences, *p* < 0.05 with differently labeled letters. **(E)** QQ plot (P) show the significantly different OTUs in the three modules, Δ: significant difference OTU. **(F)** The correlation between the screened OTU and LZM, Trypsin, LPS and α_AMS (Spearman’s *p* < 0.05: *, Spearman’s *p* < 0.01: **, and Spearman’s *p* < 0.001: ***). **(G-J)** The activities of Trypsin, LPS, α-AMS and LZM in each group were, respectively. Data are presented as mean ± standard deviation. *p < 0.05, **p < 0.01, and ***p < 0.01.

By establishing the relevant network of microorganisms, we found three microbial modules 1, 2, 7 (Figure 5A). Their species composition is shown in Supplementary Figure S2. When the composition of the feed changes, the species abundance of these three modules also changes dramatically. Compared with the control group, the relative abundance of modules 1, 2, 7 was significantly increased after feeding high starch diet, while the addition of EuO in the diet weakened the effect of high starch and decreased the relative abundance of modules 1, 2, 7 (Figures 5B–D). We also examined the intestinal activities of trypsin, lipase (LPS), and α-amylase (α-AMS) and the plasma levels of lysozyme (LZM), and found that the activities of LPS in the intestine of fish did not differ significantly among the groups (Figure 5I), while the activity of α-AMS in the GT group was significantly lower than the Z group, but the activity of α-AMS in the G1 and G2 groups increased after feeding diets containing EuO, and trypsin and α-AMS had similar changes (Figures 5G,H). In addition, the plasma levels of lysozyme in the GT group were significantly lower than those in the other groups (Figure 5J). To further understand the relationship between gut microbial changes and digestive enzymes and lysozyme, we first selected 10 significantly different OTU from these three modules (Figure 5E), and then performed a regression analysis, and found that these OTUs (OTU275, OTU328, OTU312, OTU546, and OTU272) were strongly negatively correlated with LZM activity, with OTU272 and OTU312 being strongly negatively correlated with α-AMS activity (Figure 5F). The annotation information of OTUs are in Supplementary Table S2.

## 4. Discussion

The intolerance of *M. salmoides* to high starch is widespread, but this problem has not been effectively solved due to the restriction of feed process and feed cost. In this study, we utilized a feed formulation containing 22% α cassava starch to investigate the beneficial effects of EuO on intestinal damage caused by high starch and changes in intestinal microbial structure.

Intestinal microbiota is an important part of fish intestines. Compared with mammals, the number of intestinal microflora in fish is low and fluctuates greatly (Egerton et al., 2018; Wang et al., 2018). The environment is one of the main ways in which fish acquire microorganisms and is an important factor influencing the microbial composition of the fish intestinal (Liu et al., 2021). Previous studies have shown that the composition of intestinal microflora of fish is similar to that of the water in which they live (Kim et al., 2021). In previous studies on intestinal microbiota of *M. salmoides*, it was found that the intestinal microbiota of healthy *M. salmoides* was mainly composed of Firmicutes, Proteobacteria and Fusobacteriota (Huang X, et al., 2021). However, in this study, it was found that the intestinal microbiota of *M. salmoides* was mainly composed of Firmicutes, Proteobacteria and Actinobacteriota, which was probably caused by the living environment.

Intestinal microorganisms of aquatic animals are highly sensitive to food composition (Lopez Nadal et al., 2020). Diet is thought to be a major factor affecting the composition and function of the gut and the bacteria that live in it. Previous research has shown that high-fat and high-sugar diets could alter the intestinal microbiome, resulting in dysbiosis (Tan et al., 2021). And dysbiosis of the intestinal flora could trigger local inflammation and damage the lining of the intestine. Bacteroidetes are considered to be major degraders of polysaccharides, their genomes contain a large number of polysaccharide utilization sites, and a reduction in the abundance of Bacteroidetes decreases the utilization of polysaccharides (McKee et al., 2021; Zafar and Saier, 2021). Proteobacteria contain a variety of bacteria related to enteritis. In the mouse model, it was found that innate immune disorders promote the growth of Proteobacteria, resulting in intestinal inflammation (Shin et al., 2015). Studies have shown that high starch diets may promote the accumulation of *M. salmoides* AGEs, leading to the dissociation of NF-κB and translocation to the nucleus to upregulate the transcription of pro-inflammatory factors, including TNF-α, and exacerbate the inflammatory response (Wang et al., 2023). In this study, high starch was found to cause intestinal inflammation in largemouth bass by HE staining, and high expression of NF-κB and TNFα, which are pro-inflammatory factors, was also detected. Analysis of the intestinal flora revealed that high starch could reduce the abundance of Firmicutes and Bacteroidetes, and increase the abundance of Proteobacteria and Actinobacteriota. This may be an important factor in causing intestinal inflammation. In addition, excess starch in the diet promotes the accumulation of fatty acids, which can lead to inflammation (Wang et al., 2023). In the present study, an increase in fatty acid degradation was found in the high-starch group, which may be a strategy for fish to cope with high-starch foods.

EuO is an herb that has the effect of regulating the intestinal flora of animals. In mammals, EuO extracts altered the intestinal flora structure and improved growth performance in weaned piglets (Peng et al., 2019). In fish, EuO altered the structure of the intestinal flora of *Ictalurus punctatus*, increased the abundance of Cetobacterium and Romboutsia, and improved resistance to pathogens (Zhang et al., 2022). However, it is not clear whether EUO could suppress the adverse effects of high starch. In this study, we found that the addition of EuO reduced the richness of gut microbiota, but improved the resistance of fish to pathogens and alleviated the intestinal inflammation caused by high starch. Functional analysis of microorganisms revealed that EuO significantly enhanced functions such as bacterial chemotaxis, porphyrin metabolism and flagellar assembly. Among them, flagellar synthesis and chemotactic motility are prerequisites for the initial formation of the biofilm (Liu et al., 2022). This is important for fish to resist the adverse external environment.

The study of inter-microbial correlations in the gut will facilitate the discovery of keystone species (i.e., strong correlations with multiple species), which play a key role in the stability of the gut ecosystem. Symbiotic networks are an important reflection of inter-microbial correlation analysis and have been widely used to elucidate the mechanisms of inter-microbial community interactions. Lysozyme is an antimicrobial substance widely present in fish, which not only participates in the non-specific immune response of fish and improves their resistance to bacteria and viruses, but also promotes the activity of digestive enzymes in fish, which helps digestion and absorption (Song et al., 2021). In this study, three high-abundance OTU modules (moduel1, moduel2 and Moduel7) and 10 OTUs were identified using a microbial symbiotic network. In addition, this study found that EuO increased the lysozyme content in the plasma of *M. salmoides* and that changes in lysozyme content were strongly negatively correlated with changes in the abundance of *Limnochordaceae*, *Nitrolancea*, *Lysinibacillus*, and *Hydrogenispora*. Challenge experiments showed that high starch could reduce the resistance of *M. salmoides* to pathogens, and EuO treatment increased the survival rate of *M. salmoides*.

## 5. Conclusion

In this study, it was found that high starch feeds not only cause damage to the intestine of *M. salmoides*, but also lead to negative changes in the intestinal microbiota. Specifically, high starch diets lead to intestinal damage, increased inflammatory cells and enhanced fatty acid degradation function, which may lead to decreased fatty acids in the intestine and cause decreased immunity. However, the addition of EuO alleviates intestinal inflammation, regulates fatty acid degradation, and improves biofilm formation-related functions such as Bacterial chemotaxis, Porphyrin metabolism and Flagellar assembly, thereby improving community stability and resistance to pathogens. In conclusion, EuO can maintain intestinal homeostasis and protect *M. salmoides* from intestinal damage caused by high starch diet. Therefore, EuO may be an effective protective measure against intestinal damage caused by high starch diets.

## Data availability statement

The data presented in the study are deposited in the NCBI repository, accession number PRJNA918502.

## Ethics statement

The animal study was approved by Sichuan Agricultural University. The study was conducted in accordance with the local legislation and institutional requirements.

## Author contributions

HL, FL, and HT contributed the work equally, formal analysis, investigation, visualization, and writing – original draft. BC, DC, and HL: formal analysis and investigation. HL, YG, and XH: data curation and writing – reviewing & editing. LL, PO, and XH: supervision, conceptualization, writing – reviewing & editing, and project administration. All authors contributed to the article and approved the submitted version.

## Funding

This research was supported by Sichuan Science and Technology Program (2022NZZJ0014) and Chengdu Science and Technology Project (2022-YF09-00040-SN and 2022-YF05-00636-SN).

## Conflict of interest

The authors declare that the research was conducted in the absence of any commercial or financial relationships that could be construed as a potential conflict of interest.

## Publisher’s note

All claims expressed in this article are solely those of the authors and do not necessarily represent those of their affiliated organizations, or those of the publisher, the editors and the reviewers. Any product that may be evaluated in this article, or claim that may be made by its manufacturer, is not guaranteed or endorsed by the publisher.
