# Supplementary material for: Eucommia ulmoides Oliver repairs the disorder of intestinal microflora caused by high starch in Micropterus salmoides and improves resistance to pathogens
Source: Front Microbiol. 2023 Sep 21;14:1223723. doi: 10.3389/fmicb.2023.1223723 (PMC10552156; doi:10.3389/fmicb.2023.1223723)
Supplement: Supplementary file 1 [file Data_Sheet_1.docx]

**Supplementary material**

**Table**

Table S1. Formulation and proximate chemical composition of the trial diets

| Ingredients | Starch level in diets (g.kg ^−1^) | |
| --- | --- | --- |
|  | control | starch |
| Fish meal^a^ | 490 | 490 |
| Casein^a^ | 130 | 130 |
| Soybean protein concentrate^a^ | 60 | 60 |
| Soybean oil^a^ | 30 | 30 |
| Soybean lecithin^a^ | 20 | 20 |
| Yeast extract^a^ | 8 | 8 |
| Ca(H_2_PO_4_)_2_^a^ | 10 | 10 |
| Choline chloride^a^ | 3 | 3 |
| Vitamin mixture^b^ | 8 | 8 |
| Mineral mixture^c^ | 5 | 5 |
| Carboxymethyl cellulose^a^ | 15 | 15 |
| Lysine^a^ | 1 | 1 |
| Manioc α- Starch^a^ | 0 | 220 |
| Zeolite powder^a^ | 220 | 0 |
| Proximate compositions (g. kg ^−1^, dry matter) | | |
| Crude protein | 490 | 491 |
| Crude lipid | 82 | 91 |
| Ash | 301 | 103 |
| Starch | 13 | 224 |

^a^ Supplied by Chengdu Sanwang Feed Ltd (Chengdu, China).

^b^ Vitamin Premix (mg. kg ^−1^ diet): vitamin A, 32.00; vitamin D3, 16.00; vitamin E, 351.83; vitamin K3, 30.03; vitamin C, 3288.80; vitamin B1, 19.77; vitamin B2, 60.00; vitamin B6, 36.43; vitamin B12, 24.00; niacinamide, 80.80; calcium-pan-tothenate, 75.10; folic acid, 6.73; inositol, 329.90; biotin, 32.00; L-carnitine, 102.03.

^c^ Mineral mix (mg. kg ^−1^ diet): FeSO_4_ (Fe), 70.33; MgSO_4_ (Mg), 351.33; CuSO_4_ (Cu), 8.00; ZnSO_4_ (Zn), 99.70; MnSO_4_ (Mn), 19.50; CoCl_2_ (Co), 19.37; Ca (IO_3_)_2_ (I), 50.17; Na_2_SeO_3_ (Se), 4.00.

Table S2. The abundance of OTUs in the three modules differed significantly

| OTU ID | Phylum | Class | Order | Family | Genus |
| --- | --- | --- | --- | --- | --- |
| OTU424 | Firmicutes | Clostridia | Peptostreptococcales-Tissierellales | Family_XI | *Tepidimicrobium* |
| OTU275 | Firmicutes | Limnochordia | Limnochordales | Limnochordaceae | *Limnochordaceae* |
| OTU568 | Bdellovibrionota | Bdellovibrionia | Bdellovibrionales | Bdellovibrionaceae | *Bdellovibrio* |
| OTU328 | Firmicutes | Limnochordia | Limnochordales | Limnochordaceae | *Limnochordaceae* |
| OTU312 | Chloroflexi | Chloroflexia | Thermomicrobiales | Thermomicrobiaceae | *Nitrolancea* |
| OTU236 | Firmicutes | Bacilli | Paenibacillales | Paenibacillaceae | *Paenibacillus* |
| OTU541 | Firmicutes | Bacilli | Bacillales | Bacillaceae | *-* |
| OTU446 | Firmicutes | Bacilli | Bacillales | Bacillaceae | *Ureibacillus* |
| OTU546 | Firmicutes | Bacilli | Bacillales | Planococcaceae | *Lysinibacillus* |
| OTU272 | Firmicutes | Limnochordia | Limnochordia | Limnochordia | *Hydrogenispora* |

**Figure**

**
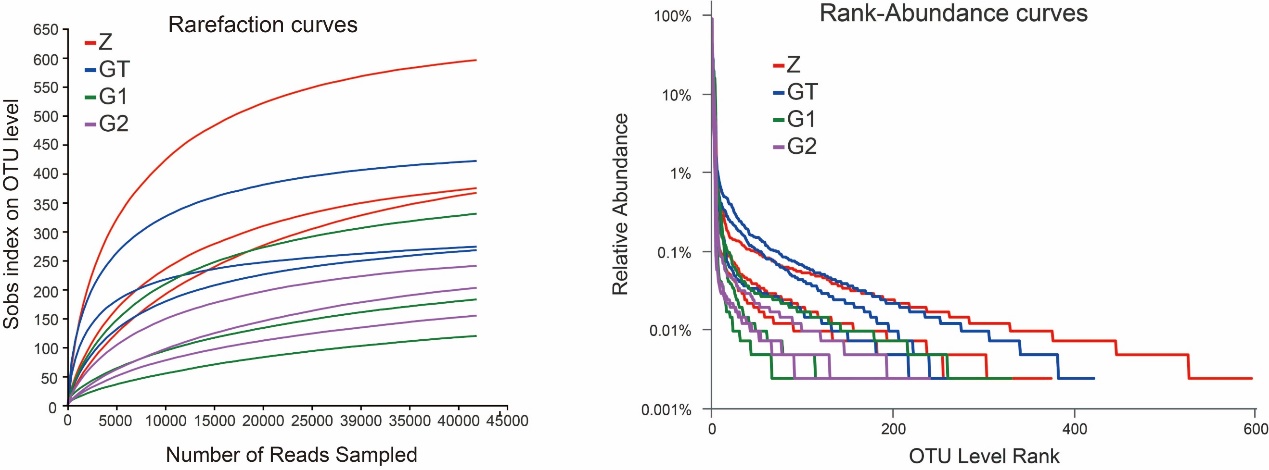
**

Fig S1. Species annotation and evaluation. A. Sobs index on OUT level. B. Rank-Abundance curve at the OTU level.


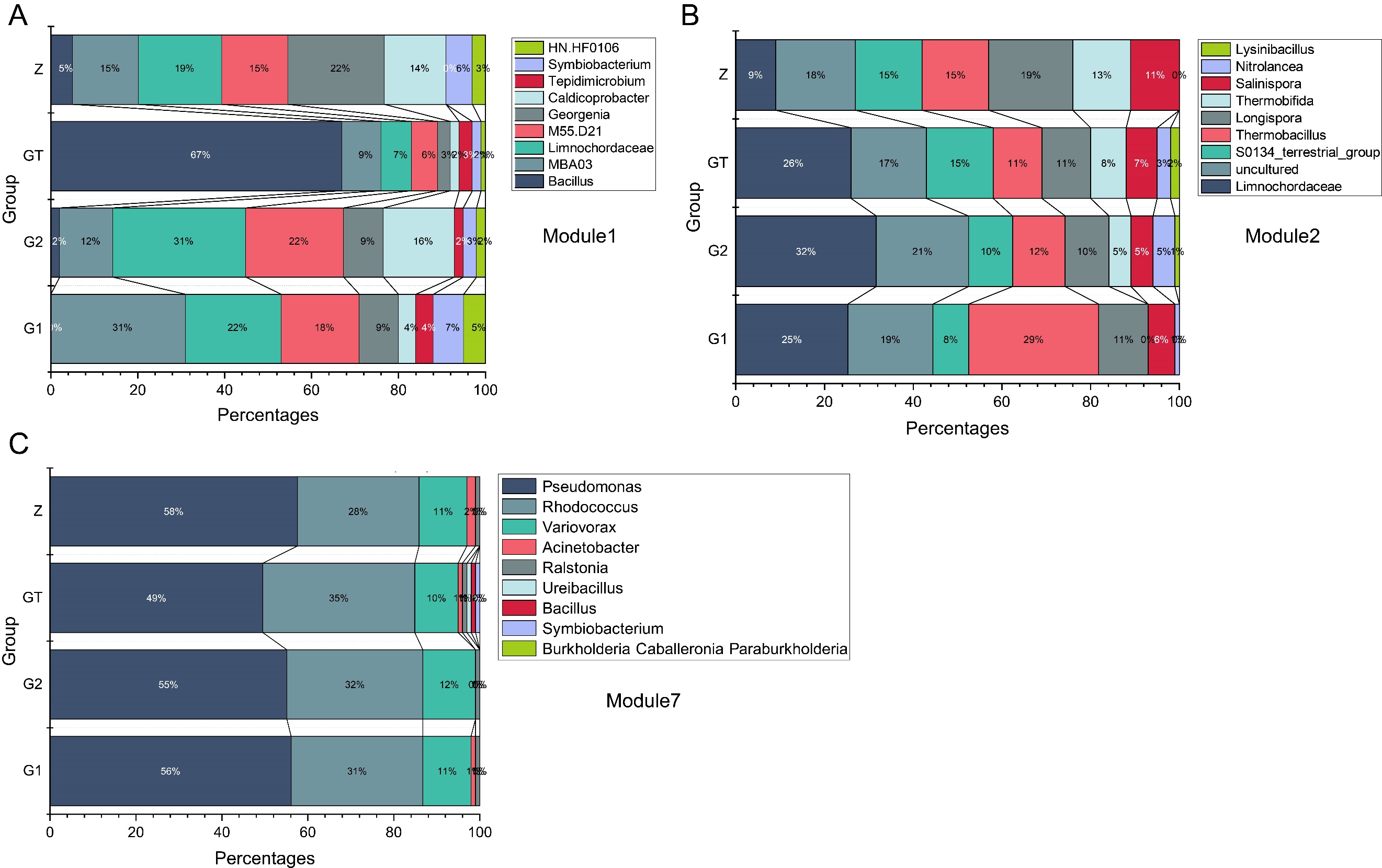


Fig S2. Species composition of module1, module2 and module3.
