# Supplementary figures and images for: Eucommia ulmoides Oliver repairs the disorder of intestinal microflora caused by high starch in Micropterus salmoides and improves resistance to pathogens
Source: Front Microbiol. 2023 Sep 21;14:1223723. doi: 10.3389/fmicb.2023.1223723 (PMC10552156; doi:10.3389/fmicb.2023.1223723)

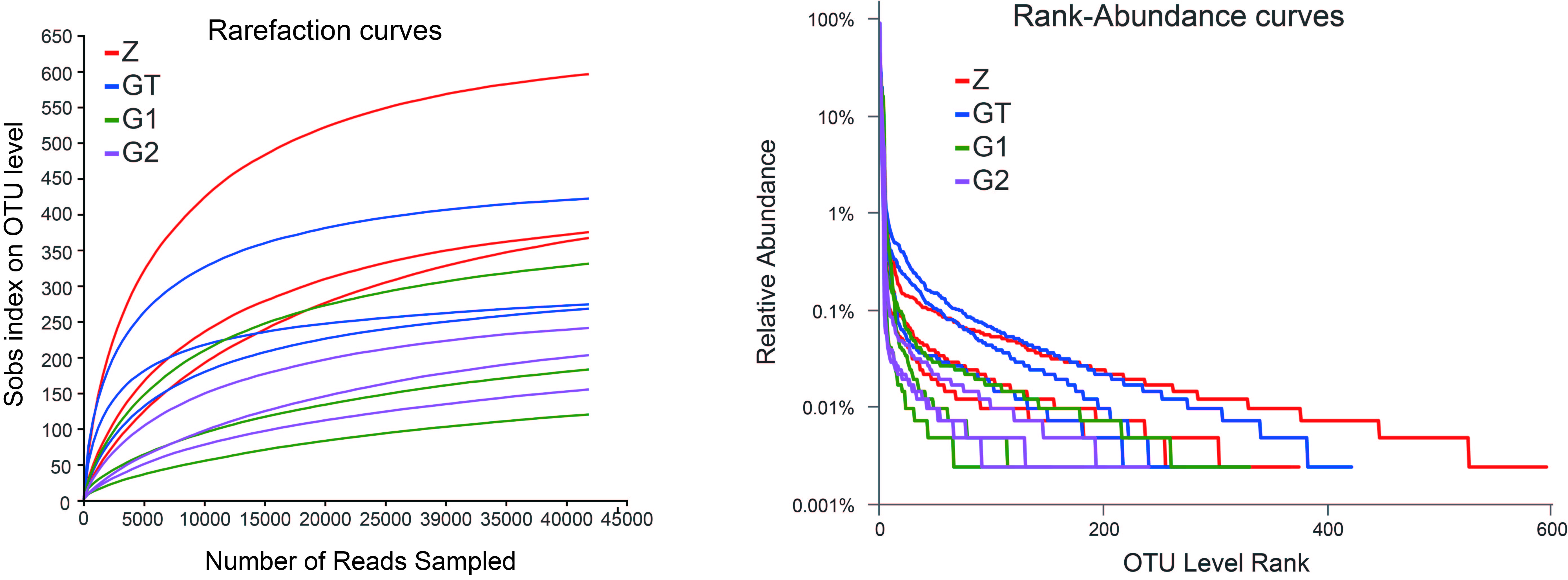

Supplement: Supplementary file 2 [file Image_1.JPEG]
